# Prenatal ultrasound diagnosis and pregnancy outcome of umbilical cord knot – debate regarding ethical aspects of a series of cases

**Published:** 2016

**Authors:** RE Bohîlțea, N Turcan, M Cîrstoiu

**Affiliations:** * ”Carol Davila” University of Medicine and Pharmacy, Bucharest, Romania; ** University Emergency Hospital Bucharest, Romania

**Keywords:** umbilical cord knot, prenatal diagnosis, Doppler, 3D ultrasound

## Abstract

True umbilical cord knot appears to be a relatively common complication that occurs in 0.3%–1.3% of all pregnancies and it is correlated with an increased incidence of SGA infants, premature birth, need for neonatal intensive care and fetal death. The aim of the article was to evaluate the incidence of the true umbilical cord knot in the University Emergency Hospital, Bucharest, for a period of 5 years and its association with premature birth, low birth weight, low Apgar score at 1 minute and the need for neonatal intensive care. By reviewing the total number of women who delivered in this unit between January 1st 2011 and December 31st 2015, the percentage of the diagnosis antepartum and intrapartum, the outcome of these pregnancies, and the reflection of this condition on the fetal status, were evaluated. During 5 years, 133 (0.71%) of 18.500 deliveries were diagnosed with true umbilical cord knot, only 16 (0.08%) cases were diagnosed by ultrasound antepartum. The mean maternal age was 34.3 years. About 30% of the studied cases (39) presented this condition at the third delivery or more. A personal history for diabetes corresponded to 27 cases (20.3%). From our database, it resulted that only 12 fetuses (10.5%) required neonatal intensive care and presented an Apgar score lower than 7 at 1 minute. Prenatal diagnosis of a true umbilical cord knot is rarely encountered and sonography skills are needed. Complementary methods such as color Doppler and 3D HD Flow are reliable for the diagnostic when true umbilical cord knots are suspected after a 2D scan. Several risk factors can guide the expectancy, such as advanced maternal age, polyhydramnios, multiparty or diabetes.

## Introduction

The occurrence of the umbilical cord knot is considered to be 0.3-1.3% of all pregnancies [**1**]. The frequency of antepartum fetal death is four to ten times higher in the fetuses who present this pathology; for all that, antepartum management of these cases is currently undetermined [**2**]. These rarely cause problems before labor, but may do so in labor. Several risk factors can raise the suspicion of the existence of this condition: advanced maternal age, multiparity, obesity, previous spontaneous abortion, chronic hypertension and gestational diabetes. Some obstetric conditions can be related to umbilical cord knot, such as genetic amniocentesis, male fetus, small fetuses, polyhydramnios, long umbilical cord and prolonged gestation. As consequences, studies reported cord accidents [**3**,**4**], fetal acidosis [**5**], low Apgar score at 1 minute, higher risk of cesarean delivery and intrauterine fetal death [**6**]. As most of the length of the cord develops prior to the end of the second trimester, most knots must occur in the early part of the pregnancy where the fetus somersaults through the loop [**7**]. The most threatening complication of the umbilical cord knot is the constriction of the knot and its reflection, the interruption of blood flow to the fetus resulting in fetal death.

True umbilical cord knot often remains undiscovered prenatally due to a lack of clinical characteristic or ultrasound functional signs except for the impact on flows of a tight knot or the incidental visualization. Prenatal diagnosis of an umbilical cord true knot is difficult through 2D ultrasound, but today, with ultrasound achievement in 3D and Doppler mode is easily possible. True knots should be distinguished from false knots. The suspicion of the presence of a true umbilical cord knot appears when a cross-section of an umbilical cord surrounded by a circular loop is observed on a gray-scale ultrasound. It is unusual for a knot to tighten, especially before the onset of labor. The confirmation requires the acquiring of color Doppler three-dimensional volume of the suspected anatomical section of the cord and by rotating the volume along the x- and y-axes until a full view of the knot is displayed. The compression of the cord by a constricted true knot can be appreciated by pulsed velocimetry Doppler of the umbilical and abnormal findings, this method being able to confirm the constricted knot [**8**]. Increased umbilical-cerebral Doppler ratio may reflect acute hypoxic compromise caused by the transitory constriction of the true umbilical cord knot with unrecognized morphologic and circulatory signs. The reasons for a true knot antenatal tightening remain unknown. Some assumptions are that the fetus itself is responsible by either tangling its feet around the cord or possibly, clutching the cord with a hand and tightening the knot [**9**].

The impact on neonatal outcome of a prenatal diagnosis of a true umbilical cord knot has not been fully evaluated, recent publication noted that cord entanglement does not contribute to prenatal morbidity and mortality in monoamniotic twin pregnancies [**10**].

Carlos Lopez RC [**11**] described the prenatal diagnosis of a true knot of the umbilical cord by using the “hanging noose” sign and the analysis of the changes in the tension of the knot related to fetal movements with 4-dimensional ultrasonography without a clear diagnosis in all circumstances.

“Hanging noose” is a sign that is considered diagnostic for a true umbilical cord knot when a transverse section of the umbilical cord surrounded by 1 of its loops is observed at ultrasound examination [**11**]. Benrit described a series of different kind of true umbilical cord knots, with its correspondents in images. Some sonographic characteristics, such as the four-leaf clover or an unusual multicolored pattern visualized on color flow imaging, have been described as being highly suspicious of true knot, but these findings have not been specific for the diagnosis [**12**].

## Materials and methods

The aim of this study was to evaluate the incidence of the true umbilical cord knot at the University Emergency Hospital in Bucharest for a period of 5 years and its association with premature birth, low birth weight, low Apgar score at 1 minute and the need for neonatal intensive care and also to determine the percentage of the diagnosis antepartum and intrapartum and the outcome of these pregnancies.

Our retrospective study included data for January 1st 2011 to December 31st 2015 collected from the statistical database of the University Emergency Hospital in Bucharest. Data on neonatal birth characteristics and perinatal outcome of fetuses diagnosed with umbilical cord knot antepartum and postpartum were also included. From the database, personal data, obstetric history was selected among the associated pathologies. Regarding the fetus, the gestational age based on the last menstrual period or by ultrasound, fetal birth weight and Apgar score at 1 minute were studied.

## Results

Of the 18 500 women who gave birth at the University Emergency Hospital in Bucharest during the studied period, 133 (0.71%) were diagnosed with true umbilical cord knot, the obtained result complying with the reported incidence by other international studies.

**Fig. 1:**
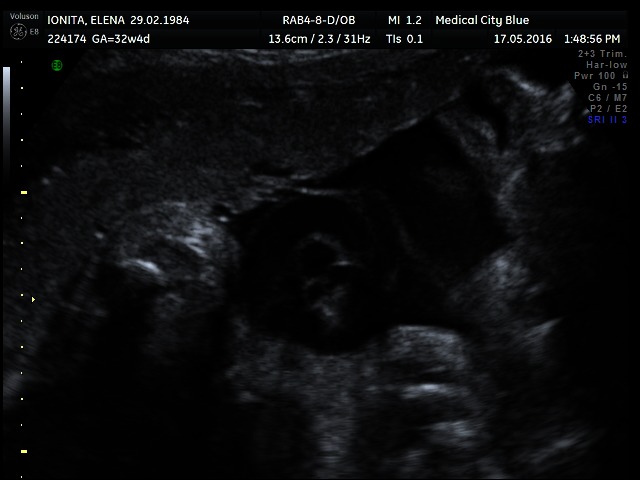
A hanging noose sign is seen on the 2-dimensional ultrasonography; a transverse section of the umbilical cord is surrounded by a loop of umbilical cord

**Fig. 2:**
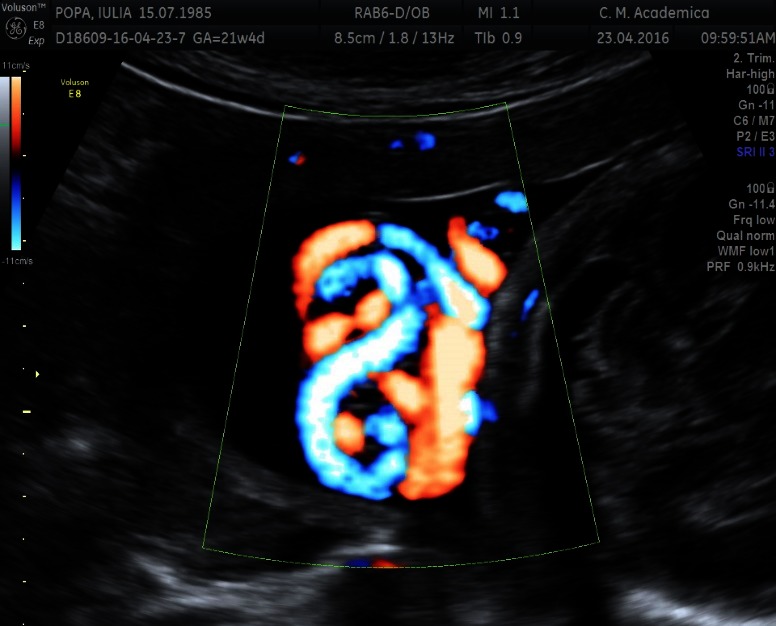
HD Flow image of a true umbilical knot

**Fig. 3:**
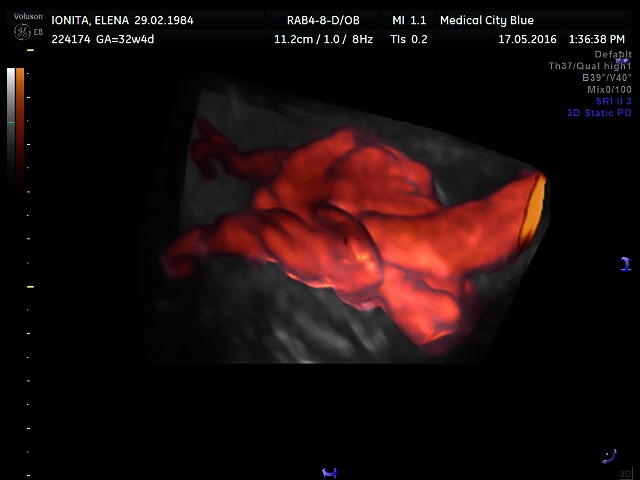
True umbilical cord knot on 3D power Doppler Glass Body

**Fig. 4:**
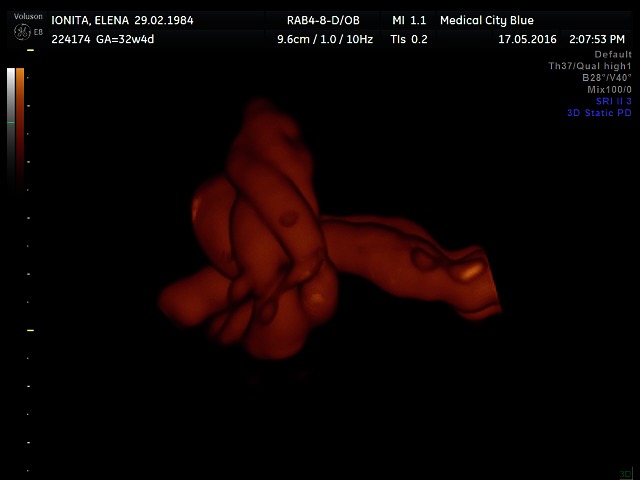
True umbilical cord knot on 3D power Doppler

**Fig. 5:**
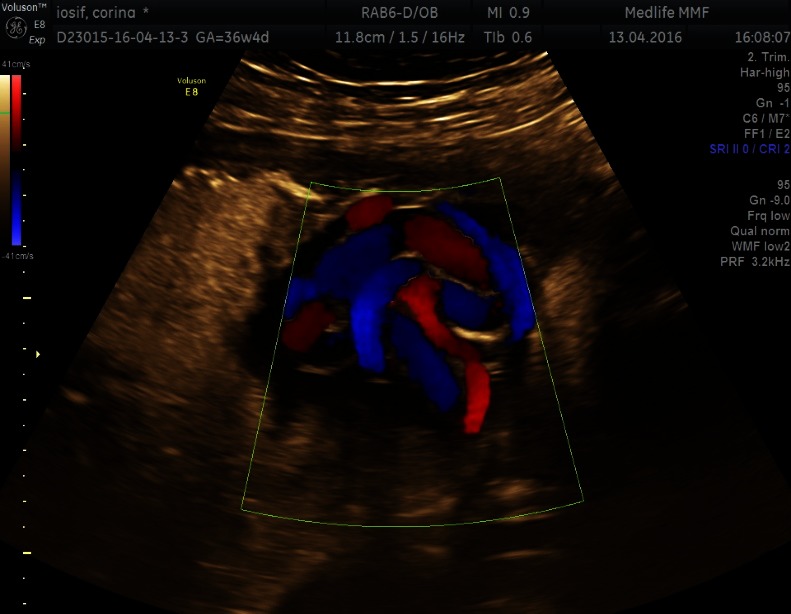
False umbilical cord knot on color Doppler

**Fig. 6:**
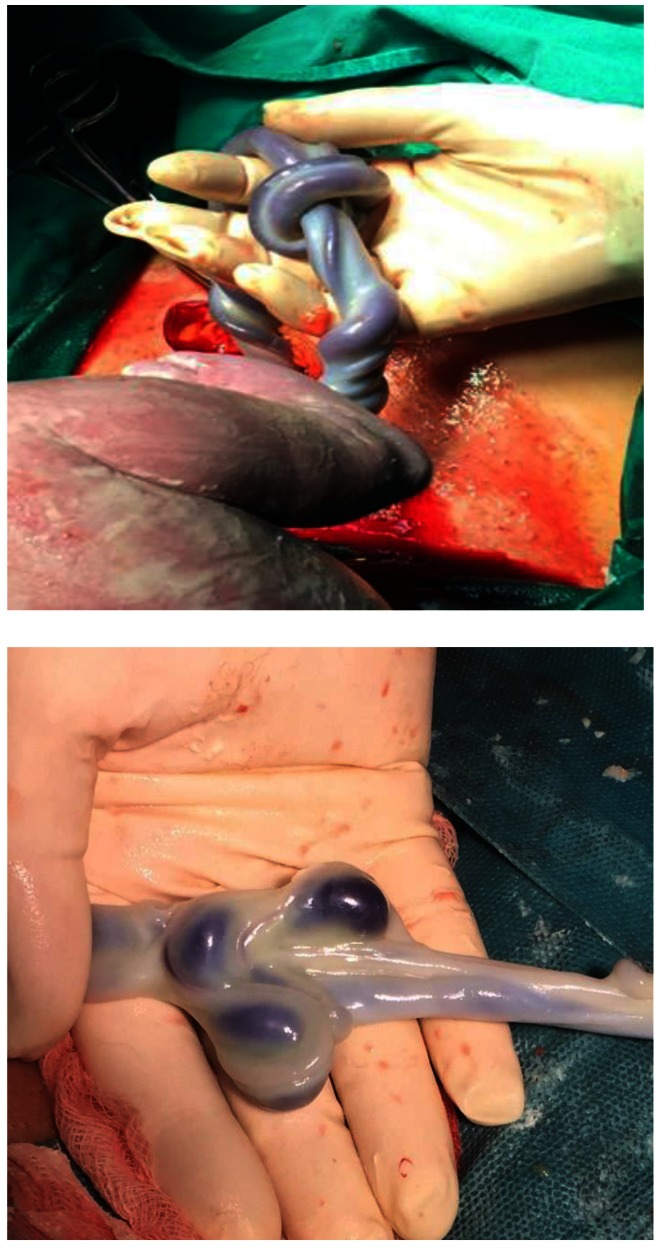
Intrapartum aspect of a true (left) and false (right) umbilical cord knot

**Fig. 7:**
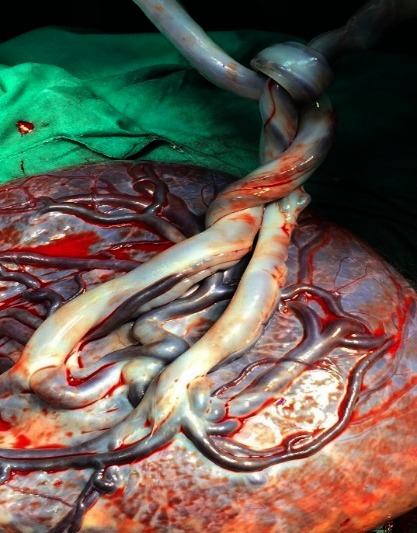
True umbilical cord knot diagnosed antepartum and confirmed intrapartum in a monochorionic monoamniotic twin pregnancy, on term birth

Only 16 cases (12% of the umbilical cord knot cases) were correctly diagnosed with umbilical cord antepartum by ultrasonography (**Fig. 1**-**4**). False knots were recorded in 4 cases (**Fig. 5**). 117 cases (88%) were diagnosed intrapartum (**Fig. 6**,**7**). The diagnosis was mainly sonography skills dependent.

**Fig. 8:**
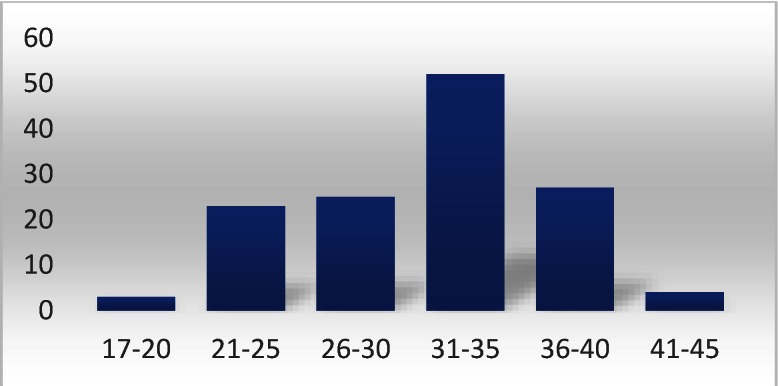
Division according to age groups

The mean maternal age was 34.3 years. Division according to age groups showed a predominance of the umbilical cord knot in women aged between 30 and 35 years, followed by women aged between 36 and 40 years (**Fig. 8**).

**Fig. 9:**
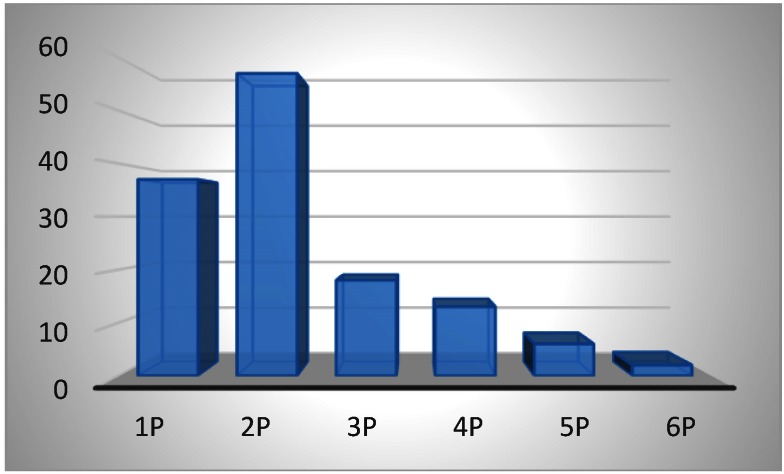
Division according to parity

Another aspect studied was the number of deliveries of each patient diagnosed antepartum or intrapartum with true umbilical cord knot and a possible association of the appearance of this condition and multiparity, considering other studies that developed this aspect as mentioned above. In our studied group, the majority of true umbilical cord knots were diagnosed in women at their second pregnancy, 42.8% (57 cases). The other 37 women presented true umbilical cord knot at their first pregnancy. About 30% of the studied cases (39 cases) presented this condition at the third delivery or more. The p-value calculated with chi square test was 0.03 < 0.05, resulting that in our study the link between multiparity and the presence of true umbilical cord knot was statistically significant (**Fig. 9**). 

**Fig. 10:**
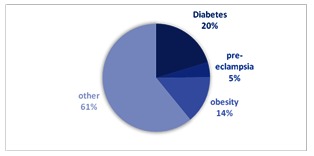
Associated pathology in umbilical cord knot cases

The personal history of each patient diagnosed with true umbilical cord knot was also analyzed in the studied period, according a special attention for diabetes, pre-eclampsia and obesity. A prevalence of diabetes of 20.3% (27 cases) was obtained in the studied group, 6 cases (4.5%) were diagnosed with pre-eclampsia and 19 (14.28%) were obese. Pre-eclampsia was considered as gestational blood pressure of at least 140/90 mm Hg and at least 300 mg of protein in the urine over 24 hours. Diabetes mellitus was defined by high blood glucose levels in oral 75-g glucose tolerance test (a jeune ≥ 92 mg/ dL; after 1 hour ≥ 180 mg/ dL; and after 2 hours ≥ 153 mg/ dL). An obese woman was considered a woman with a BMI of 30 or higher (**Fig. 10**).

**Fig. 11:**
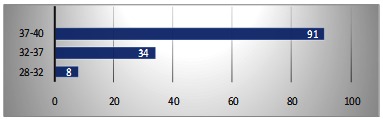
Distribution of umbilical cord knot according to the gestational age of delivery

Regarding the reflection of this condition on the fetus, the gestational age, birth weight and the Apgar score at 1 minute were analyzed. Almost 70% of the fetuses with confirmed umbilical cord knot were delivered at a gestational age of 37-40 weeks, 8 fetuses being delivered at 28-32 weeks of gestation (**Fig. 11**). Gestational age was defined based on the date of the last menstrual period or by ultrasonography.

Regarding the birth weight, 44 newborns (33%) were rated within 3000-3500 grams and from our database it resulted that only 12 fetuses (10.5%) required neonatal intensive care and presented the Apgar score lower than 7 at 1 minute. No fetal deaths were registered. 

## Discussions and Conclusions 

True umbilical cord knot is a relative frequent condition that rarely complicates the pregnancy. Various factors related to the complications of the umbilical cord require a careful sonographic and Doppler examination. 

The advanced technology offers a large opportunity to visualize detailed fetal and placental structures. Mostly benefic, there are authors who have wondered if in some cases we do not see too much [**13**]. The observation of an unusual coiling of the umbilical cord, the “hanging nose” sign on the ultrasound examination, that may indicate a true umbilical cord knot, without other specific symptoms and abnormal sonographic finding and normal Doppler measurements, generates a dilemma for the obstetrician, who alternates between the decision of communicating and informing the patient on the suspicion of a true umbilical cord knot and the risks related to this condition or not, and the way it is ethical to do it. The question that remains open is if the patient has something to gain from this information besides the anxiety that this knowledge brings and the way this will reflect on the management of the delivery. Prenatal diagnosis of a true umbilical cord knot is rarely encountered. Complementary methods such as color Doppler and 3D HD Flow are reliable for the diagnostic when true umbilical cord knots are suspected after a 2D scan. Several risk factors can guide the expectancy, such as advanced maternal age, polyhydramnios, multiparty or diabetes.
